# The ectonucleotidases CD39 and CD73 on T cells: The new pillar of hematological malignancy

**DOI:** 10.3389/fimmu.2023.1110325

**Published:** 2023-01-27

**Authors:** Xuan Jiang, Xiaofang Wu, Yuxi Xiao, Penglin Wang, Jiamian Zheng, Xiuli Wu, Zhenyi Jin

**Affiliations:** ^1^ Key Laboratory for Regenerative Medicine of Ministry of Education, Institute of Hematology, School of Medicine, Jinan University, Guangzhou, China; ^2^ Department of Pathology, School of Medicine, Jinan University, Guangzhou, China

**Keywords:** CD39, CD73, T cells, hematological malignancy, immunotherapy

## Abstract

Hematological malignancy develops and applies various mechanisms to induce immune escape, in part through an immunosuppressive microenvironment. Adenosine is an immunosuppressive metabolite produced at high levels within the tumor microenvironment (TME). Adenosine signaling through the A_2A_ receptor expressed on immune cells, such as T cells, potently dampens immune responses. Extracellular adenosine generated by ectonucleoside triphosphate diphosphohydrolase-1 (CD39) and ecto-5’-nucleotidase (CD73) molecules is a newly recognized ‘immune checkpoint mediator’ and leads to the identification of immunosuppressive adenosine as an essential regulator in hematological malignancies. In this Review, we provide an overview of the detailed distribution and function of CD39 and CD73 ectoenzymes in the TME and the effects of CD39 and CD73 inhibition on preclinical hematological malignancy data, which provides insights into the potential clinical applications for immunotherapy.

## 1 Introduction

**Figure 1:**
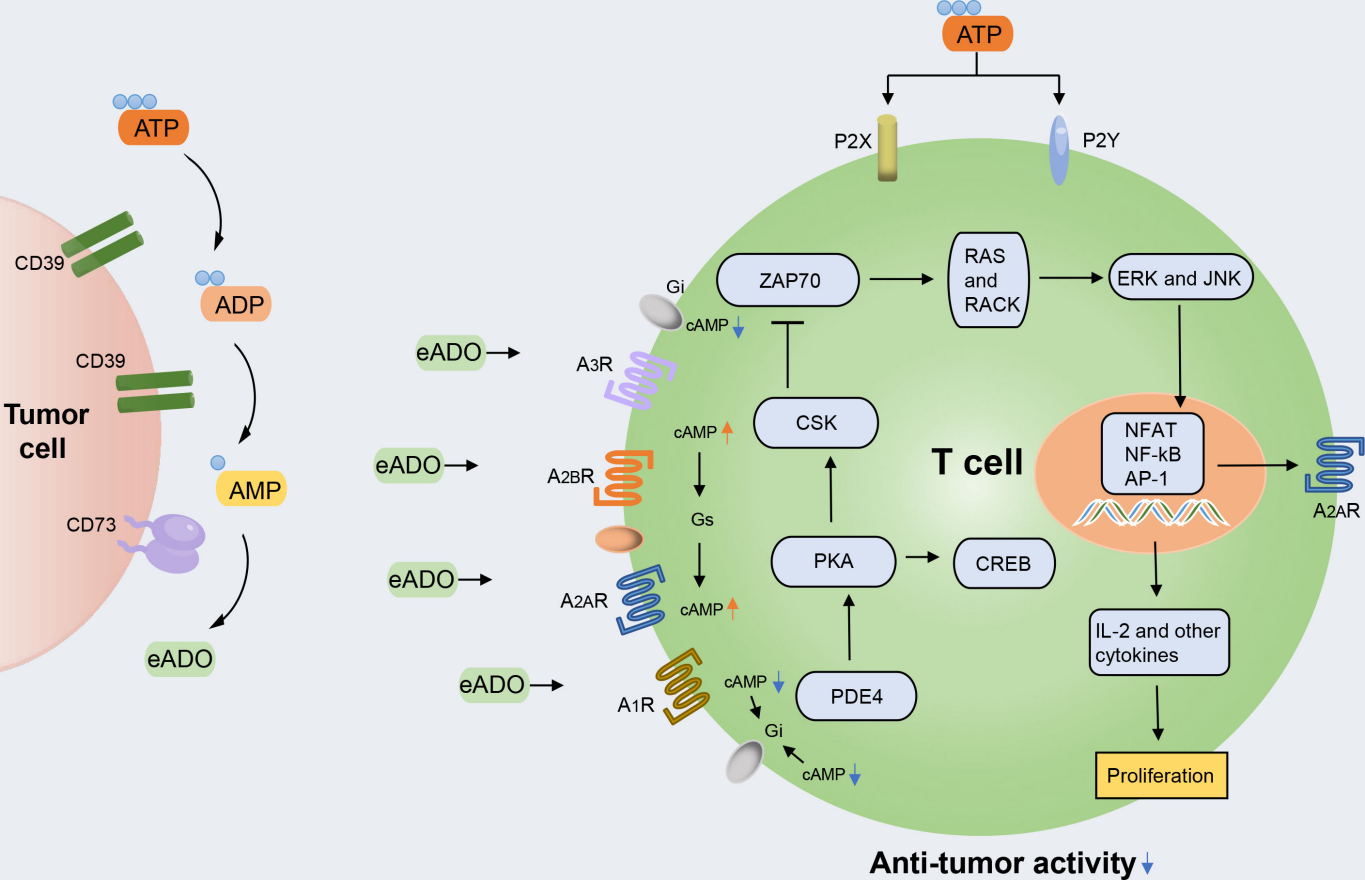
The ATP-adenosine pathway regulates immune response in the tumor microenvironment (TME). The accumulated extracellular ATP (eATP) can activate immune cell inflammation activity by stimulating type 2 purinergic receptors (P2XR and P2YR). The accumulated extracellular ADO (eADO) can bind to the downstream purinergic receptors (A_1_R, A_2A_R, A_2B_R, and A_3_R), resulting in the accumulation of cAMP. cAMP accumulation leads to protein kinase A (PKA) phosphorylation and the activation of C-terminal Src kinase (CSK), which reduces downstream LCK-dependent activation of ZAP70, extracellular signal-regulated kinase 1 (ERK1), and JNK and protein kinase C (PKC). PKA activation also activates cAMP-responsive element-binding protein 1 (CREB), which contributes to the inhibition of the major pro-inflammatory transcription factor nuclear factor-κB (NF-κB). Through this intracellular signaling pathway, the TCR-mediated activation of immune cells is counteracted.

In the tumor microenvironment (TME), unusually high extracellular adenosine concentrations promote tumor proliferation through various immunosuppressive mechanisms. Adenosine triphosphate (ATP) represents the currency for energy metabolism inside the cell. By contrast, extracellular space usually derives from passive leakage from necrotic or injured cells, enhancing inflammation, hypoxia, and cancer (1, 2). High extracellular ATP (eATP) concentrations influence cell metabolism, adhesion, and migration in acute inflammation, in which the ectonucleotidases CD39 and CD73 take part in catabolizing nucleotides and producing immunosuppressant adenosine (ADO), which are devoted to restoring homeostasis. The ATP degradation pathway proceeds through CD39, which converts eATP or ADP to AMP, and CD73, which hydrolyzes and converts AMP to ADO (3) (**Figure 1**). Although ectonucleotidases help prevent excessive inflammation and tissue damage, their contribution to generating an immunosuppressive microenvironment in tumor biology is more worrying. In hematological malignancies, the overexpression of CD39 and CD73 has been linked to increased homing to protected niches, increased survival, proliferation, and modulation of immune responses toward tolerance (4, 5). In some instances, ectonucleotidases have become reliable markers for monitoring disease and stratifying patient subsets or molecular targets for novel treatment strategies.

In this review, we discuss the structure and function of CD39 and CD73 in physiological conditions and then focus on their expression and roles in the TME of several hematological malignancies. In addition, we illustrate their potential as new targets in hematological malignancies, and the experimental findings and clinical trials of CD39 or CD73 therapies are extensively discussed.

## 2 Classic features of CD39 and CD73

The cascade starting with ATP and leading to ADO production is governed by CD39 and CD73, which affect purinergic signaling by modulating ligand availability (6). CD39 is an extracellular enzyme known as ecto-nucleotide triphosphate diphosphohydrolase 1 (ENTPDase1), which belongs to the membrane-bound extracellular nucleoside triphosphate diphosphate hydrolase family. It is an integral membrane protein depending on Ca^2+^ and Mg^2+^. Human CD39, encoded by the *ENTPD1* gene on the 10q24.1 chromosome, is a protein composed of 510 amino acids, and its molecular weight is approximately 78 kDa. CD39 contains seven heavily glycosylated N-linked glycosylation sites, 11 cysteine residues, and two transmembrane regions. These two transmembrane regions include a small cytoplasmic domain containing NH_2_- and COOH-terminal segments and a large extracellular hydrophobic domain containing five highly conserved domains known as apyrase conserved domains (ACRs) 1-5. This structure is significant for the catabolic activity of the enzyme and the maintenance of molecular structural integrity and contributes to nucleotide binding (7).

CD73, also known as ecto-5’-nucleotidase (ecto-5’-NT), is a membrane-bound glycoprotein connected by glycosylphosphatidylinositols (GPI) (8). Encoded by the *NT5E* gene located on human chromosome 6q14-21, CD73 is a protein molecule composed of 574 amino acids (according to its cDNA sequence), the molecular weight of which in its naturally purified form is 70 kDa (9). CD73 consists of three domains: the N-terminal domain with a metal binding site, the C-terminal domain in which the catalytic site is located, and the bridged α-helix domain (10). The non-covalent hydrophobic interaction at the C terminus and the binding of two zinc ions can stabilize the homodimerization of CD73 and achieve complete catalytic activity. CD73 homodimer can effectively hydrolyze AMP and convert it into ADO by opening and closing conformational cycles.

For this reason, it is also called the rate-limiting enzyme of the second step of purine nucleotide metabolism (11). ADO is a nucleoside molecule produced by the hydrolysis of ATP and is a critical signal molecule in the ATP-adenosine pathway. ADO can bind to four adenosine receptors belonging to the same G protein-coupled receptor (GPCR) family: A_1_R, A_2A_R, A_2B_R, and A_3_R. Among these, A_1_R and A_3_R are preferentially coupled to Gi protein to inhibit the action of adenylate cyclase and reduce the production of cyclic adenosine monophosphate (cAMP). However, A_2A_R and A_2B_R are generally Gs-coupled and trigger the action of adenylate cyclase and subsequently promote the production and accumulation of intracellular cAMP (12, 13). cAMP accumulation can activate both the canonical protein kinase A (PKA) and the non-canonical EPAC pathways (5). Additionally, all four adenosine receptors have been shown to induce the mitogen-activated protein kinase (MAPK) and JUN N-terminal kinase (JNK) pathways (14) (**Figure 1**).

## 3 CD39 and CD73 in the TME

### 3.1 CD39 and CD73 expressed on immune cells

**Figure 2:**
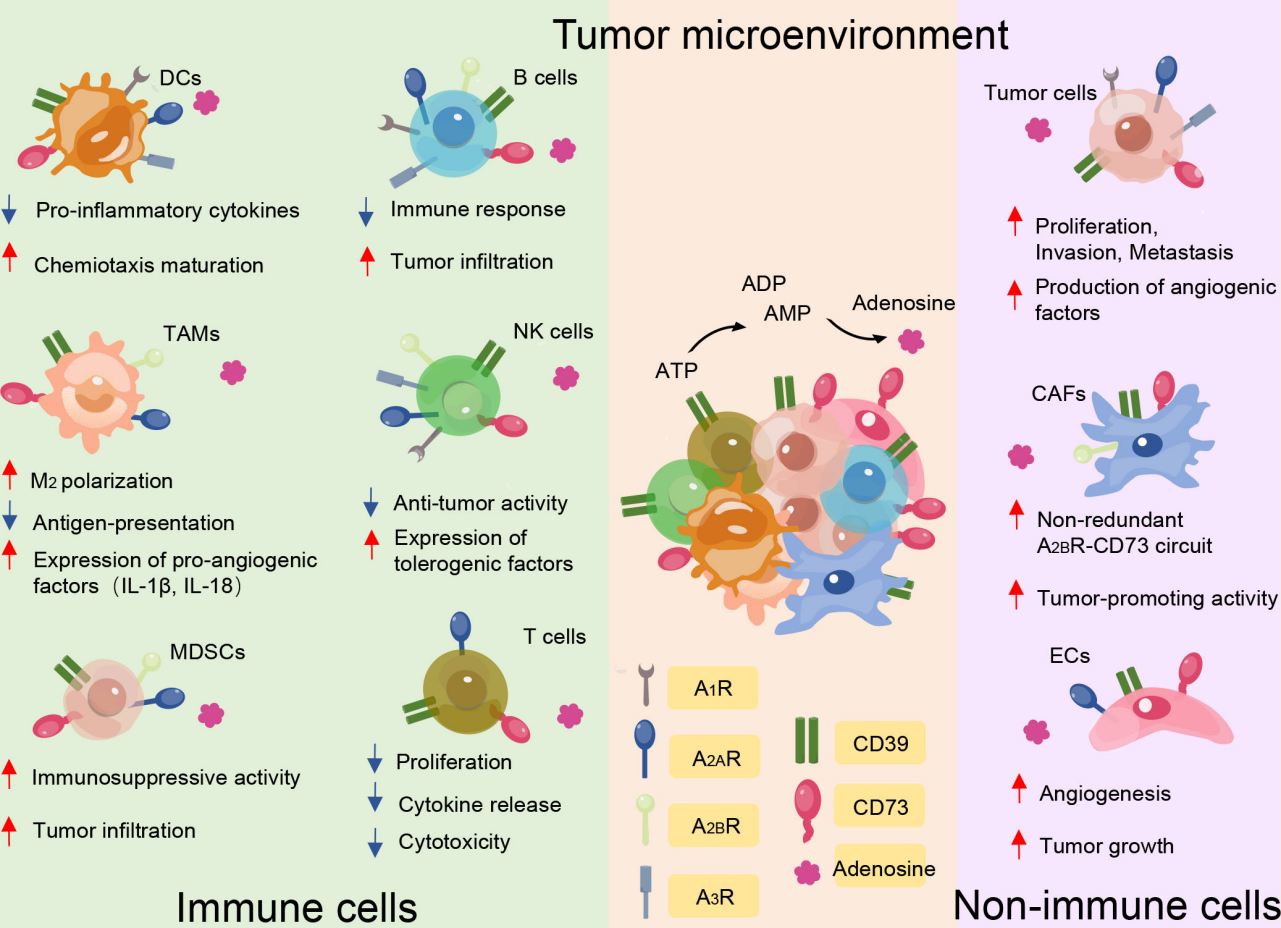
CD39 and CD73 serve as major immune suppressive mediators in the tumor microenvironment mainly through the generation of eADO. Besides the effect of ectonucleotidases on tumor cell proliferation, angiogenesis, infiltration, and metastasis, CD39 and CD73 expression by immune cells and non-immune cells impairs anti-tumor immunity by suppressing the function of protective immune cells, including T cells, B cells, natural killer (NK) cells, dendritic cells (DCs), myeloid derived suppressor cells (MDSCs), and tumor-associated macrophages, while maintaining the function of non-immune cells, including tumor cells, cancer-associated fibroblasts (CAFs), and endothelial cells (ECs). The red and blue arrows indicate whether functions are enhanced or reduced by adenosine binding to the different receptor subtypes.

Interactions between tumor cells and their immunological microenvironment are essential for the pathophysiology of lymphocytes, myeloid-derived suppressor cells (MDSCs), dendritic cells (DCs), and macrophages, which can co-express CD39 and CD73 (15) (**Figure 2**). Human B cells co-express CD39 and CD73 while the former was initially described as a B cell activation marker and expresses A_1_, A_2_, and A_3_ adenosine receptors (16, 17). It has been characterized that the phenotype and functionality of CD39^+^ human regulatory B cell (Breg) promotes Breg functions and shows high proliferative capacity while acting through adenosine generation and interleukin-10 (IL-10) secretion to immunosuppress T cells (18). CD73 is broadly expressed in human peripheral blood (PB) B cells and can also be expressed in memory B cells that develop outside of the germinal center, such as in the context of an extrafollicular reaction (19). Notably, adenosine-producing B cells produce significantly more interleukin-6 (IL-6) and IL-10, and activation of A_1_ and A_2A_ receptors promote expansion and increase the differentiation of B cells toward class-switched B cells (20). Natural killer (NK) cells belonging to the innate immune subset are involved in anti-tumor immunity and contribute to the effects of ATP through type 2 purinergic receptors (P2XR and P2YR). CD39 and CD73 expression levels in NK cells are low but increase under specific conditions. CD39 can inhibit NK cell-mediated damage and decrease interferon-γ (IFN-γ) secretion (21). Additionally, CD73 expression is virtually absent in NK cells in healthy individuals but significant in tumor-infiltrating tissues, which suggests that NK cells can exert immunosuppressive function through the production of adenosine, environmental factors permitting (22).

CD39 and CD73 also exert their pro-tolerogenic effects on myeloid compartments. CD39 and CD73 levels of MDSCs are higher in tumor patients than in healthy controls (23). A positive correlation between intratumor CD39- and CD73-expressing MDSCs and tumor stage, node involvement, and metastasis status in non-small cell lung cancer has been reported (24). In further research, MDSCs expressing high levels of CD39 and CD73 increased immunosuppressive activity *ex vivo* compared with myeloid cells present in colorectal cancer (25). Thus, MDSCs that infiltrate tumors are probably an important source of extracellular adenosine, which contributes to tumor immune escape. eATP can activate the immune system through the stimulation of P2XR7 on DCs and promote an increase of interleukin-1β (IL-1β) and interleukin-18 (IL-18) secretion (26). Furthermore, IL-1β facilitates macrophage maturation and increases cytokine production (27). Additionally, CD39 is expressed on DCs, affecting DC-driven CD4^+^ T cell activation and differentiation through NLRP3 inflammasome, which is activated by the ATP-adenosine pathway (15, 28). NLRP3 is a prerequisite for IL-1β and IL-18 production (29). Furthermore, the accumulation of adenosine can impair the normal function of DCs, the so-called immune-suppressive regulatory DCs (30). Tumor-associated macrophages co-express CD39 and the eATP receptor. Inhibiting CD39 on macrophages significantly increases their production of tumor necrosis factor-α (TNF) and interleukin-12 (IL-12), while decreasing IL-10 secretion, thus inhibiting tumor growth (31, 32). It has been suggested that these macrophages that produce ADO suppress the activation of CD4^+^ T cells *in vitro* (33). In the context of a subgroup, the classification of immune cells based on CD39 and CD73 better reflects their function.

### 3.2 CD39 and CD73 are expressed on non-immune cells

Increasing evidence has also verified that CD39 and CD73 are the key regulatory molecules in tumor development, including tumor growth, metastasis, and angiogenesis, and their suppressive effects on the immune system in the TME (15). A high density of angiogenesis can support the sustenance of tumor cell growth, and angiogenesis is also an important pathway for the distant invasion of tumor cells.

CD39 is highly expressed on cancer-associated fibroblasts (CAFs) in ovarian cancer and pancreatic cancer (34). In a mouse model of chronic pancreatitis and fibrosis, it was shown that CD39-deficient mice develop significantly limited fibrosis. Additionally, tissue and plasma levels of anti-fibrotic IFN-γ significantly increased (35). These results suggest a role for CD39^+^ CAFs in promoting parenchymal fibrosis in pancreatic tissue (34). Elevated CD73 activity correlates strongly with high CAF abundance in colorectal cancer tissues (36). Furthermore, in a mouse model with ovarian cancer, a previous study demonstrated that CD73 on CAFs promotes tumor immune escape (37). ATP is well known to modulate a variety of processes linked to endothelial cell activation and increase the intracellular levels of Ca^2+^, which induces cytoskeletal rearrangements. In addition, ATP is released by endothelial cells during changes in flow or after exposure to hypoxic conditions, activating P2YR and promoting the release of vessel relaxation (38). In the TME, the expression of CD39 in the vascular system, especially endothelial cells, can promote tumor growth by scavenging eATP and promoting angiogenesis (39). In melanoma, lung carcinoma, and colon tumors, suppressed tumor growth in CD39-deficient mice has been associated with decreased angiogenesis; CD39 co-expression with CD73 in endothelial cells will ultimately generate adenosine, which promotes angiogenesis (34). Indeed, CD73-mediated adenosine and A_2A_ signaling in endothelial cells have been shown to promote angiogenesis in a variety of experimental conditions, including during tumorigenesis (40) (**Figure 2**).

## 4 CD39 and CD73 are expressed on different T cell populations in the TME

**Figure 3:**
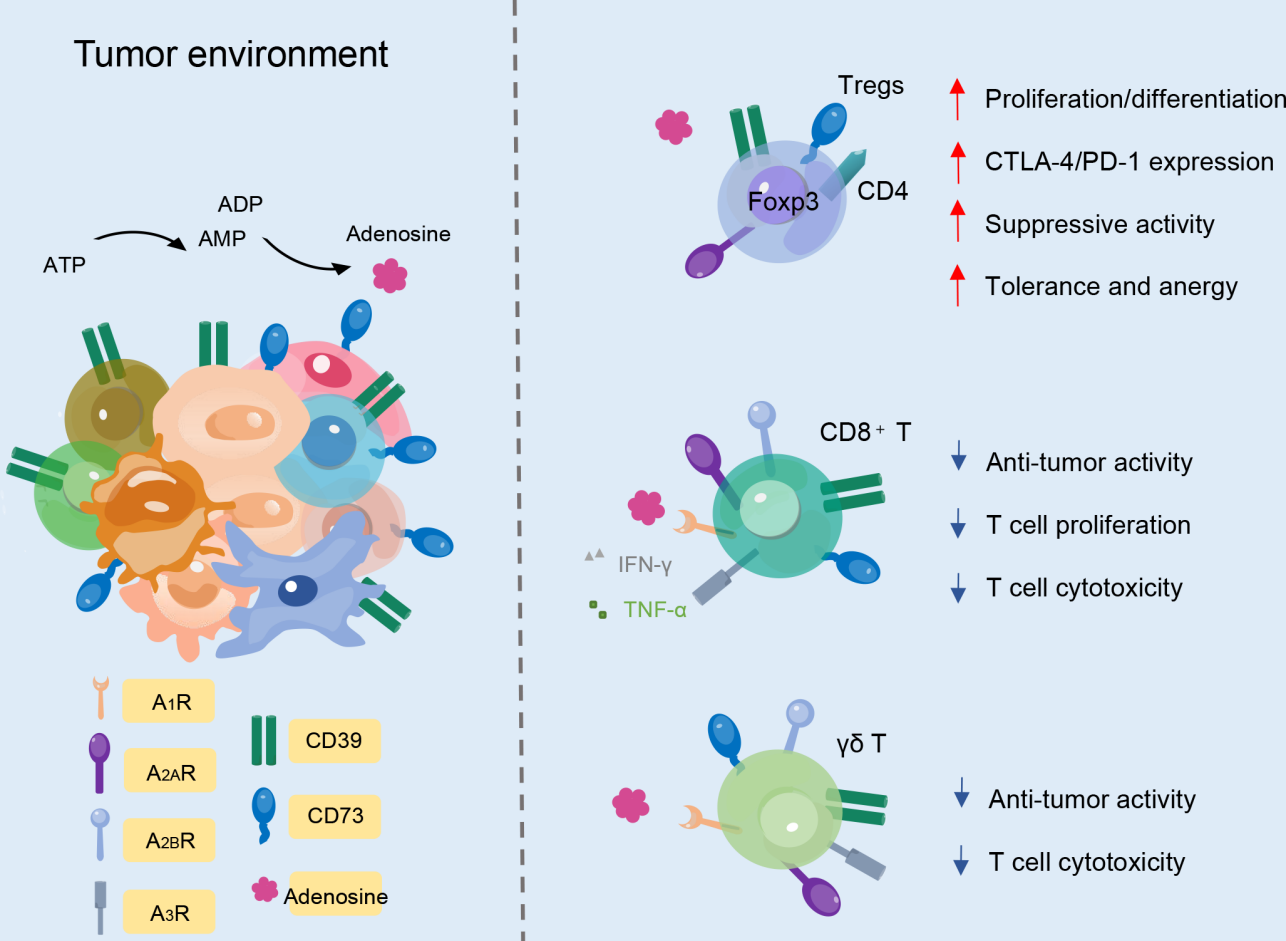
CD39 and CD73 expression on different T cell subtypes. In Foxp3^+^CD4^+^ Treg cells, CD39 and CD73 improve immunosuppressive activity. In CD8^+^ T cells, ectonucleotidases decrease cytotoxicity, proliferation, and anti-tumor activity. Likewise, CD39 and CD73 expression on γδ T cells can also decrease anti-tumor activity and T cell cytotoxicity.

The immortality of malignant cells demonstrates the host anti-tumor immune responses’ failure and induces an immunosuppressive microenvironment in which they can freely grow and expand. It has been shown that adenosine concentration is significantly increased in the TME, and a variety of immune cells, especially T cell subsets, are involved in the immunosuppression process (**Figure 3**). In effector T cells, after adenosine receptor activation, type I protein kinase A (PKA) and its C-terminal Src kinase (CSK) phosphorylation are activated to inhibit SRC family tyrosine kinases LCK and FYN. This attenuates the activation of transcription factors that are downstream of T cell receptor (TCR) activation, including NFAT, nuclear factor-κB (NF-κB), and AP-1. TCR activation increases A_2A_Rs through NF-κB-dependent induction (41). The generation of high local concentrations of adenosine by CD39 and CD73 leads to potent immunosuppression *via* the impairment of T cell activation and function, with simultaneous enhancement of regulatory T cells (Tregs) (42). Hence, the ability to block adenosine generation by inhibiting the enzymatic activity of CD39 and CD73 provides a direct line of attack on adenosine-mediated immunosuppression, and the ATP-adenosine pathway functions as a critical modulator of innate and adaptive immunity with the TME.

### 4.1 CD39 and CD73 are expressed on Treg cells

In human PB, approximately one-third of CD4^+^ T cells and a small proportion of CD8^+^ T cells express CD39 (15). On the contrary, CD73 is expressed by less than 50% of CD8^+^ T cells and by less than 10% of CD4^+^ T cells (15). Adenosine in turn modulates Treg function. Tregs play an indispensable role in maintaining immunological unresponsiveness to self-antigens, and counteraction of the immunosuppressive features of the TME is an attractive strategy for cancer treatment. ADO produced by CD39 and CD73 through the ATP-adenosine pathway can regulate the function of Tregs, activate receptors on Tregs to promote proliferation, and increase the expression of immunosuppressive receptors to enhance immunosuppressive function (43). In the TME, the aggregation of Tregs is associated with high CD39 expression, which promotes adenosine accumulation, tumor growth, and angiogenesis (44, 45). Compared with traditional Tregs, CD39^+^ Tregs show more vital inhibitory ability (46). Studies also have shown that CD39^+^ Tregs specifically suppress Interleukin-17 (IL-17) production to some extent, preventing the transdifferentiation of Tregs into T helper 17 (Th17) cells or endowing already differentiated Th17 cells with an immunosuppressive phenotype. Additionally, inhibition of human CD73 can reduce immunosuppression mediated by Tregs (47).

### 4.2 CD39 and CD73 are expressed on CD8^+^ T cells

Some studies have suggested that CD8^+^ T cells expressing CD39 and CD73 also show regulatory characteristics. Meanwhile, CD39 is potentially involved in mediating the suppressive abilities of tumor-infiltrating CD8^+^ Tregs (48). The isolated CD39^+^CD8^+^ T cells from tumor-infiltrating lymphocytes (TILs) can inhibit T cell proliferation *in vitro*, which mediates tumor invasion, and display a gene signature of exhaustion (49). CD8^+^ T cells express a high frequency of CD39 in solid tumors and non-solid tumors, which affects their normal cytotoxicity and ability to secrete cytokines (50). The phenotypes of exhaustion mean that the production of TNF-α, IFN-γ, and interleukin-2 (IL-2) cytokines decreases, accompanied by the upregulation of co-inhibitory receptors, including programmed cell death 1 (PD-1), cytotoxic T-lymphocyte-associated protein 4 (CTLA4), lymphocyte-activation gene 3 (LAG3), T cell immunoreceptor with Ig and ITIM domains (TIGIT), and T-cell immunoglobulin mucin-3 (TIM-3) (48, 51, 52). IL-6 and transforming growth factor-β (TGF-β) are additional factors that contribute to the upregulation of CD39 on CD8^+^ T cells and subsequently potentiate the immunosuppressive activity in the TME. Beyond the TME, CD39^+^CD8^+^ T cells are also abundant in invaded lymph nodes and metastases and in the peripheral circulation lymphoid organs (51, 53). Interestingly, the expansion of the CD39^+^CD8^+^ T cell population in the blood is associated with clinical responses to anti-PD-1 therapy (53).

### 4.3 CD39 and CD73 are Expressed on γδ T Cells

Human γδ TCR-expressing cells constitute 1–5% of total T cells in the PB and play an indispensable role in the immune system. γδ T cells belong to the non-conventional lymphocyte family though they can produce many cytokines, such as IFN-γ, and act cytotoxically (54). γδ T cells are composed of different subpopulations with different functions. Recent research has shown that activated murine γδ T cells co-express CD73 and CD39 and display immunosuppressive functions, while most resting γδ τ cells do not constitutively express CD39 (55). CD39 has been identified as a marker of regulatory γδ T cells (15, 55). In murine lymph nodes, the CD25^+^CD39^+^ γδ T cell population can suppress the proliferation of αβ T cells *in vitro* (55). In the TME, CD39^+^ γδ T cells of invasive mouse pancreatic tumors are upregulated, together with other immunosuppressive factors, and support tumorigenesis by inhibiting αβ T cell proliferation (56). Vγ9Vδ2 T cells are a subset of γδ T cells in the peripheral circulation and function by detecting self and pathogen-associated phosphoantigens (pAgs). Normally, these cells do not express CD39 or CD73 but can upregulate CD39 upon TCR stimulation. Gruenbacher et al. proved that CD39 dephosphorylates pAgs, which specifically activate Vγ9Vδ2 T cells, rendering them inactivate at stimulating γδ T cells, and thus revealed a previously unrecognized immunoregulatory role of CD39 (57). CD73 is expressed in more than 90% of peripheral γδ T cells (58). In a study of CD73 deficient mice, CD73 proved essential for γδ T cell development and might participate in its regulatory function (58). γδ T cells express different levels of CD73 before and after their activation, and the level of CD73 expression correlates with the pro- and anti-inflammatory activities of γδ T cells in Th17 autoimmune responses (59). Researchers have found that CD73-expressing γδ T cells are much more potent at converting AMP to adenosine than all other CD73^+^ immune cell types (59).

## 5 The role of ectonucleotidases CD39 and CD73 in hematological malignancy

### 5.1 Acute myeloid leukemia

Acute myeloid leukemia (AML) is a progressive myeloproliferative malignant tumor, which is mainly characterized by abnormal proliferation of primitive and immature myeloid cells in the bone marrow (BM) and PB (60). It has been shown that there is an abnormally high CD39 expression in Treg cells in patients with AML (35). Nicolas Dulphy et al. found that compared with healthy people, the proportion of Tregs in the circulation of AML patients increased, and the frequency of CD39 decreased (36). However, the percentage of CD39^+^ Tregs did not decrease, which suggests that the function of CD39 in Tregs of AML patients could be maintained. At the same time, only a few patients and healthy people expressed CD73 in the Tregs, and the frequency was deficient. The increase of Treg subsets indicated that there is an overall immunosuppressive environment in tissues and BM in patients with AML (61, 62).

It has been suggested that CD39^+^CD8^+^ T cells can be used as a potential marker of exhaustion in patients with AML (63). In a study by Brauneck et al., TIGIT^+^CD73^-^CD8^+^ from AML patients showed a distinct characteristic, both in PB and BM. These cells were divided into PD1^+^TIGIT^+^CD73^-^CD8^+^ T and CD39^+^TIGIT^+^CD73^-^CD8^+^ T cell subsets. As the disease progressed, the proportion of PD1^+^TIGIT^+^CD73^-^CD8^+^ T cells gradually increased, and this was maintained in remission (63). The latest study suggested CD39 could be used as a marker of poor treatment response and prognosis in patients with AML. Aroua et al. graded the fold enrichment of the CD39 expression cells in AML patients after chemotherapy and found that the disease-free survival rate of the ‘high CD39 ratio’ group was significantly worse than that of the ‘low CD39 ratio’ group (64). Moreover, when the focus was on patients under 60, this survival disadvantage was more significant, indicating that CD39 could be used as a prognostic marker of adverse response to chemotherapy in AML (64). The drug blocking the inhibition of CD39 activity can not only block the mitochondrial metabolic reprogramming related to AraC resistance but also significantly enhance its cytotoxicity and sensitivity to AML cells *in vivo* and *in vitro* (64, 65). Additionally, Franziska Brauneck et al. found that γδ T cells in patients with AML expresses high levels of CD39 and PD-1, TIM-3, TIGIT, and other immunosuppressive receptors, which is similar to that of CD8^+^ T cells but higher than that of CD4^+^ T cells (66). In further analysis, the researchers found that CD39 expression on Vδ1 T cells is significantly increased and significantly co-expressed with PD-1, TIM-3, and TIGIT, which shows further depletion characteristics (66).

Similarly, CD73 is also closely related to T-cell depletion in patients with AML and can be used as an essential target (67). The frequency of CD73 expression in CD8^+^ T cells of newly diagnosed AML patients is significantly lower than that of healthy controls. This suggests that the downregulation of CD73 expression is phenotypically related to T cell depletion, and the expression of CD73 on CD8^+^ T cells is increased significantly after complete remission. Therefore, the low expression of CD73 on CD8^+^ T cells is associated with a high burden of leukemia (67). Contrary to the long-recognized negative immune regulation of ATP-adenosine signal in tumor tissue and the increase of CD73 associated with poor prognosis, the researchers found that the expression of CD73 on CD8^+^ T cells in patients with AML is related to the enhancement of immune response and has a higher function (67). On the other hand, CD73^-^CD8^+^ T cells express high levels of inhibitory receptors, such as PD-1, TIGIT, and immunosuppressive molecules, and have the ability to produce cytokines, including IL-2, TNF-α, and IFN-γ, is decreased, thus increasing the likelihood of apoptosis (67). Therefore, understanding the specific distribution pattern of CD73 in each cancer type or disease state is very important for the optimal design of clinical studies of cancer treatment of CD73 (67, 68).

### 5.2 Chronic lymphocytic leukemia

Chronic lymphocytic leukemia (CLL) is the most common type of leukemia in adults and is characterized by the proliferation and progressive accumulation of functionally deficient B cells in PB, BM, and lymphoid tissues (69). The clinical course of the disease is highly variable, and some patients have a good prognosis and a long survival time, while others can rapidly develop invasive lymphoma or leukemia (70). To better differentiate prognostic subsets, novel biological parameters have been added to clinical staging systems, and TME appears to play a critical role in genesis and progression. The expression level of CD39 on CLL cells is significantly higher than that of normal lymphocytes, and the levels of CD39^+^CD4^+^ T and CD39^+^CD8^+^ T cells in PB are also significantly higher (71). Compared with CD39^low^ T cells, the time-to-first treatment of CLL patients with CD39^high^ T cells is significantly shorter, which indicates that the expression of CD39 on CD4^+^ T cells is closely related to the more advanced stage of the disease and that CD39 plays a role in the invasion of the disease (71, 72). In addition, the number of CD39^+^CD4^+^ T cells increases in CLL patients with poor prognostic markers, which is associated with a shorter initial treatment time. In addition, the frequency of CD39^+^CD4^+^ T cells in CLL patients with cytogenetic abnormalities with poor prognosis is also similar to that in patients with normal- or low- or moderate-risk cytogenetic abnormalities (73). Above all, the data suggest that CD39^+^CD4^+^ T cells are associated with a poor prognosis in patients with CLL (73). In patients with CLL, the increase of Tregs has also been associated with disease progression, and the unique proportion of CD39^+^ Tregs subsets is related to the disease stage of CLL (74). However, compared with healthy controls, Foxp3^+^ and Foxp3^-^CD39^+^CD4^+^ T cells in CLL are increased, and the levels of these two subsets are related to the severity of CLL. This suggests that the expression of Foxp3 on CD39^+^CD4^+^ T cells has no additional predictive value for the prognosis of CLL patients (73). The results referred to above were obtained from a cross-sectional study, so it is not clear whether CD39 expression on T cells increases with the deterioration of the disease.

CD73 expression may also be related to the prognosis of CLL. M. Kicova et al. showed that high CD73 expression is related to the significant shortening of the overall survival time of CLL patients (75). This was the first time that researchers have directly proven the effect of CD73 expression on the survival of patients with CLL. In addition, CD73 expression has been found on B cells in CLL patients, and Serra et al. found that high CD73 expression is associated with more aggressive clinical behavior, which is characterized by large CLL clones and poor prognosis (75, 76). Therefore, further research is needed to determine the effect of CD73 expression in patients with progressive disease.

### 5.3 Multiple myeloma

Multiple myeloma (MM) is the second most common hematological malignancy and is characterized by abnormal proliferation of clonal and terminally differentiated B cells in the BM. Owing to the heterogeneity of its disease progression and the changes in the bone marrow microenvironment, most patients have a recurrence, and the prognosis of different patients is very different (77, 78). Therefore, individualized treatment of MM is critical. In patients with malignant MM, the number of CD39^+^ Tregs is increased and they participate in the inhibition of the Th17 response. Additionally, they are used as a myeloma cell promoter that produces IL-17, especially in myeloma-permitted BM environments (79, 80). The appearance of activated CD39^+^ Treg cells and BM resident CD39^+^ Tregs may represent the early changes caused by malignant MM cells, thus promoting the clinical progress of MM (79).

In addition, Rui Yang et al. detected the expression of CD39 on CD8^+^ T cells of MM patients. Interestingly, similar to CD8^+^ TIL cells related to antigen-specific depletion, these CD39^+^CD8^+^T cells can also co-express PD-1 (81). In addition, Arghya Ray et al. found that targeted CD73 therapy, alone or in combination with an immune stimulant TLR-7 agonist, can enhance the activity of MM-specific CD8^+^ cytotoxic T cells, which is a promising new strategy to restore patients’ anti-MM immunity (23, 82). In the BM of patients with MM, the expression of CD39 on γδ T cells is significantly increased, especially on Vδ1 T cell subsets (66). Moreover, CD39 is often co-expressed with inhibitory receptors, such as TIGIT, PD-1, and TIM-3 on γδ T cells, which suggests that γδ T cells may be in a state of depletion. Therefore, targeted CD39 has potential application value in activating and enhancing the cytotoxicity of γδ T cells.

### 5.4 Diffuse large B-cell lymphoma

Diffuse large B-cell lymphoma (DLBCL) is the most common subtype of aggressive non-Hodgkin’s lymphoma and can occur *de novo* or as a result of the transformation of indolent lymphoma (83). Owing to the heterogeneity of DLBCL, approximately one-third of patients still have a poor prognosis. The latest study found that the ATP-adenosine axis can inhibit the activity of CD8^+^ T cells, and the combination of PD-1 and CD73 can define more dysfunctional CD8^+^ T cell subsets (84). Targeting of the PD-1/PD-L1 (programmed cell death-ligand 1) immunosuppressive pathway combined with CD73 inhibitors may provide additional clinical benefits and partially overcome primary and secondary drug resistance to PD-1/PD-L1 blockade, as well as put forward a strong theoretical basis for precise immunotherapy and further the development of CD73 immunotherapy strategies for DLBCL patients.

## 6 Clinical study of the ectonucleotidases CD39 and CD73 in tumor immunity

The specific expression pattern of the ectonucleotidases CD39 and CD73 make them capable of serving as markers to selectively tag leukemia cells and deliver therapeutic agents while limiting off-targets. Additionally, as they operate in a coordinated cascade of events, the inhibition of one of them is sufficient to block the downstream processes. Hence, their intervention opens the possibility of modulating immunosuppression.

Recent studies have shown that blocking CD39 and CD73 can not only prevent the accumulation of adenosine but also restore anti-tumor immunity by stabilizing extracellular pro-inflammatory ATP (23). As a drug target for cancer, various drugs against CD39 have entered clinical trials (**Table 1**). CD39 inhibitors, including ARL67156 and POM-1, are effective in animal models of follicular lymphoma, sarcoma, or mouse melanoma, resulting in the partial overcoming of poor T cell response to stimulation, enhanced response to chemotherapeutic drugs, and inhibition of tumor growth, respectively (47). After administration of CD39 inhibitor ARL67156, eATP in tumors increases, which promotes the recruitment of dendritic cells and CD4^+^ and CD8^+^ cells producing IFN-γ and simultaneously promotes the immune control of autophagy-deficient tumors (85). Considering the delicate balance between eATP and extracellular adenosine in regulating the immune response in TME, CD39-guided therapy may affect tumor-immune interaction in other aspects (23). In addition to monotherapy, some preclinical studies have shown that there is a synergistic blocking effect between the release of immunosuppressive tumor microenvironment and anti-PD-1/PD-L1 resulting from targeting of the ATP-adenosine pathway (including CD39 and CD73) (85). Preclinical studies have also shown that the synergistic effect of targeted CD39 antagonist IPH5201 and PD-L1 checkpoint inhibitors have better complete regression and improved survival than PD-L1 inhibitors alone (86).

**Table 1:** Antagonists of CD39 currently in clinical trials.

Clinical trial identifier	Phase	Start date	Status	Cancer type (population, N)	Interventions and combination	Primary outcome measures	Secondary outcome measures
NCT00002652	II	November 01, 1999	Completed	MM, Plasma cell tumor (N≈unknown)	Suramin	Not provided	Not provided
NCT02724163	III	January 8, 2016	Recruiting	AML (N≈700)	Mitoxantrone, fludarabine, gemtuzumab ozogamicin	DLTs, EFS, RFS	AEs, PK, CR, CIR, DCR, EFS, OS
NCT03829254	I/II	January 30, 2019	Recruiting	Advanced cancer, lymphoma, solid tumor (N≈94)	NUC-7738	DLTs, MTD, ORR, DoR, DCR, DoSD, PFS	ORR, DoR, DCR, DoSD, PFS
NCT02514083	II	July 31, 2015	Active, not recruiting	CLL, SLL (N≈29)	Fludarabine	Safety, efficacy	Not provided
NCT03884556	I/Ib	March 16, 2019	Active, not recruiting	Solid tumor, lymphoma (N≈56)	TTX-030, pembrolizumab	Safety, DLTs, MTD, RP2D	Anti-tumor activity, Cmax, PK, CD39 expression
NCT04425655	II	June 3, 2020	Recruiting	AML (N≈27)	Fludarabine	ORR, CR, CRi	Safety, CR rate, OS, LFS, EFS
NCT04261075	I	January 7, 2020	Active, not recruiting	Advanced solid tumors (N≈57)	IPH5201 (alone), durvalumab, oleclumab	Safety, ECG	DC, Cmax

ADAs, anti-drug antibodies; AEs, adverse events; ALL, acute lymphoblastic leukemia; AML, acute myelocytic leukemia; CIR, cumulative incidence of relapse; Cmax, maximum concentration; CR, complete response; CRi, incomplete count recovery; DC, disease control; DCR, disease control rate; DLTs, dose-limiting toxicity; DoR, duration of response; DoSD, duration of stable disease; ECG, electrocardiogram; EFS, event-free survival; LFS, leukemia-free survival; MM, multiple myeloma; MTD, maximum tolerated dose; ORR, objective response rate; OS, overall survival; PFS, progression-free survival; PK, pharmacokinetics; RFS, relapse-free survival; RP2D, recommended phase 2 dose.

Durvalumab, humanized anti-human PD-L1 monoclonal antibody; fludarabine, CD39 antagonist; gemtuzumab ozogamicin, CD33 Inhibitor; IPH5201, CD39 antagonist; mitoxantrone, CD39 antagonist; NUC-7738, CD39 antagonist; oleclumab, anti-CD73 monoclonal antibody; pembrolizumab, humanized anti-human PD-1 monoclonal antibody; suramin, CD39 antagonist; TTX-030, CD39 antagonist.

CD73 can also be expressed in normal cells, and for this reason, therapy targeting CD73 (such as an anti-CD73 monoclonal antibody) is often considered a non-specific therapy (87). Interestingly, studies have proved that CD73 is highly effective in targeted therapy for cancer (**Table 2**). Antagonists targeting CD73 often combine with other immune checkpoint blockers to improve the prognosis for cancer patients. Tests of CD73 small molecule inhibitor AB680 in pancreatic cancer patients have shown that it can effectively restore T cell proliferation, cytokine secretion, and suppressed cytotoxicity (88). Consistent with the previously reported anti-tumor effect of immune checkpoint blockers combined with CD73 targeted drugs, the use of AB680 combined with PD-1 blocking *in vitro* can overcome the inhibitory effect of adenosine on human T cells and enhance the anti-tumor activity of drugs and the anti-tumor effect *in vivo* (88). In addition, in the breast cancer model, anti-CD73 antibodies partially prevent lung metastasis in mice (89). Currently, a therapeutic anti-CD73 antibody MEDI9447 is also in clinical trials with patients with solid cancer (NCT02503774; NCT03611556) (90).

**Table 2:** Antagonists of CD73 currently in clinical trials.

Clinical trial identifier	Phase	Start date	Status	Cancer type (population, N)	Interventions and combination	Primary outcome measures	Secondary outcome measures
NCT03249636	Not provided	August 9, 2017	Not provided	ALL (N≈50)	Flow cytometric analysis	Expression of markers in ALL	Not provided
NCT03454451	I	April 25, 2018	Recruiting	NHL, solid tumor (N≈378)	CPI-006, ciforadenant/pembrolizumab	DLTs, MDL	AUC, Cmax
NCT04668300	II	November 26, 2020	Recruiting	Sarcoma (N≈75)	Oleclumab, durvalumab	RR, EFS	PFS, RR, OS, AEs
NCT05227144	I	January 6, 2022	Recruiting	R/R MM (N≈48)	ORIC-533	RP2D, safety, tolerability	Cmax, AUClast, PK
NCT02503774	I	July 24, 2015	Active, not recruiting	Solid tumor (N≈190)	MEDI9447, MEDI4736	Safety, SAEs	OR, DoR, DC, PFS, OS
NCT02754141	I/II	June 21, 2016	Completed	Solid tumor (N≈234)	BMS-986179, nivolumab	AEs, SAEs	ORR, DoR, PFSR, ADAs, DF, etc.
NCT03381274	I/II	May 8, 2018	Active, not recruiting	NSCLC (N≈43)	MEDI9447, AZD4635	Safety, RR	DoR, DC, PFS, OS, OR, etc.
NCT03267589	II	June 16, 2018	Completed	Ovarian cancer (N≈25)	MEDI9447, durvalumab, tremelilumab	DCR	PFS, OS, RR, DoR
NCT03835949	I	July 16, 2019	Active, not recruiting	Solid tumor (N≈36)	TJ004309, atezolizumab	MTD	II agent, Antitumor activity, etc.
NCT04672434	I	November 19, 2020	Recruiting	Solid tumor (N≈100)	Sym024, Sym021	AEs, MTD/MAD	OR, SD, TTP, AUC, Cmax, Tmax, etc.
NCT05174585	I/II	August, 2022	Not yet recruiting	Solid tumor (N≈62)	JAB-BX102, pembrolizumab	DLTs, AEs, ORR, DOR	PK, ORR, DoR, DCR, PFS
NCT04572152	I	January 18, 2021	Recruiting	Solid tumor (N≈195)	AK119, AK104	AEs, DLTs	ORR, DCR, Cmax, Cmin, ADAs
NCT04940286	II	September 28, 2021	Recruiting	Solid tumor (N≈30)	Oleclumab, durvalumab	RR, AEs	Not provided
NCT04989387	I	October 4, 2021	Recruiting	Solid tumor (N≈230)	INCA00186, retifanlimab, INCB106385	Safety, tolerability, DLTs, RDE	ORR, DCR, DoR, Cmax, CL, etc.
NCT05431270	I	June 23, 2022	Recruiting	Solid tumor (N≈38)	PT199, anti-PD-1 monoclonal antibody	MTD	RR, PK

ADAs, anti-drug antibodies; AEs, adverse events; AUC, area under the curve; CL, clearance; CLL, chronic lymphocytic leukemia; Cmax, maximum concentration; Cmin, minimum observed concentration; DC, disease control; DCR, disease control rate; DF, degree of fluctuation or fluctuation index; DLTs, dose-limiting toxicity; DoR, duration of response; EFS, event-free survival; MDL, maximum dose level; MTD, maximum tolerated dose; MM, multiple myeloma; NHL, non-Hodgkin lymphoma; NSCLC, non-small-cell lung cancer; OR, objective response; ORR, objective response rate; OS, overall survival; PK, pharmacokinetics; PFS, progression-free survival; PFSR, progression-free survival rate; RDE, recommended dose for expansion; RP2D, recommended phase 2 dose; RR, relative risk; SAEs, serious adverse events; SD, stable disease; Tmax, time to reach maximum concentration; TTP, time to progression.

AK104, anti-PD-1/CTLA-4 bispecific antibody; AK119, CD73 antagonist; atezolizumab, PD-1/PD-L1 inhibitor; AZD4635, A2AR antagonist; BMS-986179, CD73 antagonist; ciforadenant, A2A antagonist; CPI-006, CD73 antagonist; durvalumab, humanized anti-human PD-L1 monoclonal antibody; INCA00186, CD73 antagonist; INCB106385, anti-A2AR/A2BR bispecific antibody; JAB-BX102, CD73 antagonist; MEDI4736, humanized anti-human PD-L1 monoclonal antibody; MEDI9447, CD73 antagonist; nivolumab, PD-1 inhibitor; oleclumab, CD73 antagonist; ORIC-533, CD73 antagonist; pembrolizumab, humanized anti-human PD-1 monoclonal antibody; PT199, CD73 antagonist; retifanlimab, PD-1 inhibitor; Sym021, humanized anti-human PD-1 monoclonal antibody; Sym024, CD73 antagonist; TJ004309, CD73 antagonist; tremelilumab, anti-CTLA-4 monoclonal antibody.

## 7 Conclusion

Although ectonucleotidases CD39 and CD73 represent promising targets for novel therapeutic strategies, most current therapeutic strategies come from solid tumors. They involve hematological malignancies in which they can act as disease and prognostic markers and, to some extent, directly contribute to leukemia progress and expansion. The proper design of clinical trials incorporating a comprehensive biomarker strategy will be paramount for robustly impacting tumor-immune interactions and regulating the suppressive TME. As discussed previously, there are additional combination regimens that can synergize with CD39 or CD73 blockade to provide potential benefits to patients. It is worth noting that some key issues remain unaddressed, including determining the consequences of targeting CD39, CD73, and adenosine receptors on extracellular ATP levels, evaluating the activity of the dual targeting of CD39 and CD73, and developing reliable methods to measure extracellular adenosine levels in the TME.

## Author contributions

XLW and ZYJ contributed to the outline and revision of the manuscript; XJ wrote the initial manuscript draft; XFW prepared the figure; JMZ and YXX organized the literature for the manuscript; PLW and XJ revised the final draft. All authors read and approved the submitted manuscript.
